# N-acetyl cysteine in ovulation induction of PCOS women underwent intrauterine insemination: An RCT

**Published:** 2017-04-10

**Authors:** Tahereh Behrouzi Lak, Masoomeh Hajshafiha, Fariba Nanbakhsh, Sima Oshnouei

**Affiliations:** *Department of Obstetrics and Gynecology, Urmia Reproductive Health Research Center, Urmia, Iran.*

**Keywords:** N-acetyl cysteine, Polycystic ovary syndrome, Intrauterine insemination, Ovulation induction

## Abstract

**Background:**

N-acetyl cysteine (NAC) was proposed as an adjuvant to clomiphene citrate for ovulation induction in patients with polycystic ovary syndrome (PCOS) without clomiphene citrate resistance.

**Objective:**

To evaluate the effect of NAC on pregnancy rate in PCOS patients who were candidates for intrauterine insemination.

**Materials and Methods:**

In this randomized clinical trial, 97 PCOS women aged 18-38 years were enrolled in two groups, randomly. For the case group (n=49), NAC (1.2 gr)+ clomiphene citrate (100 mg) + letrozole (5mg) were prescribed daily from the third day of menstruation cycle for five days. The control group (n=48) had the same drug regimen without NAC. In order to follicular development, recombinant human follicle stimulating hormone (r-hFSH; Gonal-F®) was injected on days of 7-11 menstrual cycles in all participants. When the follicle size was 18mm or more, 10000 IU human chorionic gonadotropin was injected intramuscular and the intrauterine insemination was performed after 34-36 hr.

**Results:**

There were not significant differences between study groups regarding mean endometrial thickness (p=0.14), the mean number of mature follicles (p=0.20), and the pregnancy rate (p=0.09).

**Conclusion:**

NAC is ineffective in inducing or augmenting ovulation in PCOS patients who were candidates for intrauterine insemination and cannot be recommended as an adjuvant to CC in such patients.

## Introduction

Polycystic ovary syndrome (PCOS) is the most common cause of anovulation among reproductive- age women (1, 2). It is characterized by hyperandrogenism, insulin resistance, and chronic anovulation and affects 5-10% of women in reproductive age (3-5). Clomiphene citrate (CC) is recommended as the first treatment strategy to induce ovulation in these patients (6). Patients who do not ovulate while receiving even 150 mg CC are classified as CC-resistant patients (7). In these cases, other treatment regimens are recommended such as the co-administration of metformin and CC (that its impact is questioned), Gonadotropin (which has high costs and side effects such as multiple pregnancies and ovarian hyperstimulation syndrome), and ovarian drilling by diathermy (8, 9). However, a recent Cochrane review revealed that whereas metformin was associated with improved clinical pregnancy and ovulation rate, it did not improve live birth rates when used alone or in combination with CC or when compared with CC (10). 

Therefore, there is a need for developing therapeutic selections for treating the women with PCOS. Aromatase is a cytochrome P450 enzyme which converts androstenedione and testosterone to estrone and estradiol by hydroxylation (11). Aromatase Inhibitors particularly letrozole have been prescribed for ovulation induction in patients who do not respond to CC (12). With respect to the performance of aromatase, the enzyme inhibitor medicines inhibit the estrogen production and consequently, the pituitary gland is stimulated to secrete follicle-stimulating hormone (FSH) and ovarian follicle grows larger followed by FSH (13). 

N-Acetyl Cysteine (NAC) with the chemical structure of sulfhydryl groups is derived from L- amino acid cysteine and the first time were used as a mucolytic drug in some respiratory diseases such as chronic bronchitis (14). NAC decreased blood levels of homocysteine by increasing glutathione synthesis which is an antioxidant (15). On the other hand, NAC significantly reduces the serum testosterone level, insulin resistance, and serum lipids (16). It was shown that the combination of NAC and CC enhance ovulation rate and pregnancy rate in CC-resistant PCOS patients (17). By administering NAC with CC in PCOS patients without CC resistance, a considerable improvement in the ovulation rate, serum estrogen and progesterone, endometrial thickness, and pregnancy rate were observed (15, 18). According to the results of Salehpour study, NAC is as a safe and well-tolerated adjuvant to CC for induction of ovulation can improve the ovulation and pregnancy rates in PCOS patients (19). 

We aimed to evaluate the effect of NAC with a combination of two inductions of ovulation drugs in PCOS patients who were candidates for intrauterine insemination (IUI), to assess the pregnancy rate.

## Materials and methods

This randomized clinical trial was performed on 100 PCOS women candidates intrauterine insemination (IUI) who referred to Kosar Department of Obstetrics and Gynecology, Shahid Motahhari Hospital, Urmia University of Medical Sciences, Urmia, Iran. After obtaining the informed constant, the participants were randomized using closed envelopes (A and B) in two groups: The case group (received NAC) and control group (without NAC). Inclusion criteria were PCOS women who were IUI candidate, age 18-38 years, having two out of three criteria of chronic oligo-or anovulation, clinical or laboratory signs of hyperandrogenism, PCOS sonographic findings (based on the Rotterdam criteria (20), also the presence of normal laboratory tests of thyroid, prolactin, and normal hysterosalpingography and a normal transvaginal ultrasound. Exclusion criteria included, the presence of ovarian cyst, FSH> 10 IU/L, and patients with ovarian hyperstimulation syndrome (OHSS) and male infertility. 

The sample size was calculated based on a level error of 5% and a power of 80% to see a difference between two groups. The estimated sample size was 84 patients (42 patients in each group), but for reaffirmation 100 patients were selected by the simple random method. Three patients in the case group and six patients in controls because of discontinuation of treatments did not receive allocated intervention.

The case group were received 100 mg CC (Clomid©, Hoechest Marion Russel, Cairo, Egypt) and 5 mg letrozole (Novartis Pharma Services, Basel, Switzerland) plus 1.2 gr NAC (Sedico, Cairo, ARE) daily, from day 3 to 7 of the menstrual cycle for one cycle. NAC was given to the subjects in the form of powder inserted in small pockets to be diluted into one standard glass of water and taken orally in two daily divided doses.

Then 75 IU of recombinant FSH (r-hFSH; Gonal-F, Merck, Serono, Germany) was injected subcutaneously from the 7^th^ of menstrual cycles and continued to 11^th^ of the cycle. On the 12^th^ day of the menstrual cycle, patients were monitored by transvaginal ultrasound examination to evaluate the mean follicular diameter and the endometrial thickness. In the presence of at least one follicle with 18-20 mm in size, 10000 IU human chorionic gonadotropin (hCG; Profasi, Serono, Switzerland) was injected intramuscularly and 36-40 hr after hCG injection, IUI was performed. The serum β-hCG level was measured on the 16^th^ day after hCG injection. In the control group, the process was same as above, but without the NAC. 

Finally, the treatment duration until the presence of mature follicle, number and size of mature follicles, endometrial thickness, the timing of hCG injection, and pregnancy, miscarriage, multiple pregnancies, and ectopic pregnancy were compared in two groups. Meanwhile, transvaginal ultrasound was performed using 7.3 MHz Probe/Fukuda Denshi. Clinical pregnancy was defined as the presence of a gestational sac on ultrasound, as confirmed by the presence of a fetal heart rate. Secondary outcomes were related to assessing of abortion, OHSS, and multiple pregnancies. 

**Ethical considerations**

This study was approved by the ethics committee of Urmia University of Medical Sciences (Ref. number: Ir.UMSU.rec.1393.179). Subjects were informed that their participation was voluntary and written consent was obtained from all participants.

**Statistical analysis**

For continuous variables, data were presented as means±SD and for categorical variables, as the number and frequency. The comparisons of variables between the two groups were made using the Fisher’s Exact test for categorical variables and independent samples *t*-test for continuous variables. Kolmogorov-Smirnov test for normality quantitative data was reviewed. Students’ *t-*test was used for normally distributed data. Statistical analysis was performed using -Statistical Package for the Social Sciences, version 17.0, SPSS Inc, Chicago, Illinois, USA (SPSS) version 17. All the cut-off for statistical significance presumed 0.05. 

## Results

**Figure 1:**
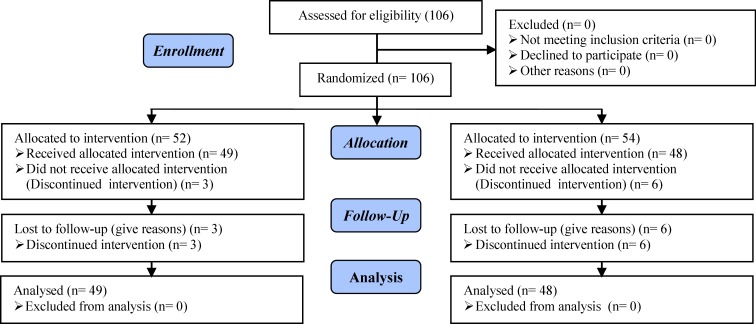
CONSORT Flow diagram

Totally, there were 106 patients in both groups at the start of this study but three of whom from the case group and 6 women from controls were excluded due to the discontinue of intervention. Therefore, 97 patients continued our study: Case group (n=49) and control group (n=48) (Figure 1). Table I shows the demographic characteristic of study participants. There were no statistically significant differences in age, body mass index, duration of infertility, the mean of luteinizing hormone (LH) and FSH, type of infertility, the mean of mature follicles, and mean number of Gonal-F in two groups (Table I, II).

The mean number of mature follicles was 2.10±0.87 in the case group and 1.85±1.03 in the control group. There was no significant difference between two groups regarding the mean number of mature follicles (p=0.20), the mean endometrial thickness on the day of hCG administration by transvaginal sonography (p=0.25), the mean day of hCG administration (p=0.47), and the mean day of IUI performing (p=0.63) (Table II). In the case group (n=49) with 49 patients, pregnancy test was positive in 16 patients (32.7%), but pregnancy test was negative in 33 patients (67.3%). Also, in the control group with 48 patients; pregnancy test was positive for 9 (18.8%) women and negative among 39 (81.2%) women. 

According to Fisher test, there was no significant difference between two groups regarding pregnancy rate (p=0.09). Besides, one case of abortion, one ectopic pregnancy (EP), and one twin pregnancy have been reported in the experimental group (Table II). No manifestations of ovarian hyperstimulation syndrome (OHSS) were reported in two groups. 

**Table I:** Demographic characteristic of study participants

**Variables**		**Case group (n=49)**	**Control group (n=48)**	**p-value**
Age (years)*		27.53 ± 4.16	27.14 ± 4.49	0.64
Body mass index (kg/m^2^)*		27.23 ± 2.90	27.84 ± 2.89	0.28
Duration of infertility(year)*		4.26 ± 3.27	3.40 ± 2.20	0.17
Serum FSH level (mIU/mL) *		6.51± 1.89	6.68± 2	0.66
Serum LH level(mIU/mL) *		9.19± 5.73	8.60± 6.05	0.67
Type of infertility n (%)				
	Primary	39 (75%)	43 (79.6%)	0.36
	Secondary	13 (25%)	11 (20.4%)	

Students’ *t*-Test, Chi-square Test

FSH: Follicle-stimulating hormone

LH: Luteinizing hormone

* Data presented as Mean±SD

**Table II:** Assessed quantity variable after intervention among two group

**Variables**	**Case group (n=49)**	**Control group (n=48)**	**p-value**
No. of mature follicles*	2.10±0.87	1.85±1.3	0.20
No. of GONAL-F injection*	4.25±1.72	4.33±2.27	0.83
Endometrial thickness (mm)*	8.15±0.85	8.04±1.4	0.25
Day of hCG administration*	14.04±1.84	14.29±1.82	0.47
Day of IUI performing*	15.93±1.74	15.87±2.68	0.63
Pregnancy rate**	16(32.7% )	9(18.8% )	0.09
Miscarriage rate**	1(6.2%)	0(0.0%)	0.62
EP rate**	1(6.2%)	0(0.0%)	0.62
Twin pregnancy **	1(6.6%)	0(0.0%)	0.62

Students’*t*-Test, Fisher’s Exact Test

EP=Ectopic pregnancy

* Data presented as Mean±SD.

** Data presented as n (%).

## Discussion

To the best of our knowledge, this is the first published study comparing the effect of NAC in PCOS women who were candidates for IUI. The meta-analysis was conducted to assess clinical benefits of NAC among women with PCOS. A total of eight randomized controlled trials with 910 women compared the effects of NAC with placebo or metformin in women with PCOS. NAC significantly improved rates of live birth and spontaneous ovulation compared to placebo in women with PCOS (21). However, we found no evidence of effects of NAC on improving pregnancy rate and spontaneous ovulations. 

Also, in the present study, the endometrial thickness on the day of hCG administration and pregnancy rate were not a significant difference in two groups. While in the study from Salehpour *et al*, the number of follicles larger than 18 mm and the mean of endometrial thickness in the group who received CC and NAC (for 6 wk) were significantly higher than those who only received CC. As well as the rate of ovulation and pregnancy in the study group who received CC and NAC was significantly greater than in the group receiving CC. Finally, they concluded that adding NAC to CC as a non-harmful adjuvant improve the ovulation rate and increases pregnancy rates (19). 

Like Salehpour et al study, Rizk et al have stated in patients who received NAC in combination with CC, ovulation and pregnancy rates were significantly increased and no cases of OHSS has been reported (22). Nasr studied the outcomes of NAC administration on CC-resistant PCOS patients who underwent unilateral laparoscopic ovarian drilling. In his study, the group receiving NAC had a significantly high rate of ovulation, pregnancy and live birth rate while the abortion rate was significantly low (23). Thakker *et al* showed that the pregnancy, ovulation and live birth rates were high in the received NAC group compared with the placebo group (21). Millea study stated that NAC administration with a dose of 1200 mg improves ovulation and pregnancy rates in PCOS patients which can be due to a decrease in insulin resistance (24).

Also, Oner et al performed a comparison study on the effects of metformin and NAC in PCOS patients, they concluded that both NAC and metformin leads to decrease in BMI, hirsutism, menstrual irregularities, levels of fasting insulin and free testosterone (25). A study on repeated failure of pregnancy concluded that the concurrent use of NAC with folic acid compared to taking folic acid alone, allow to progress further 20 wk of pregnancy and significantly increase live birth rate (26). Abu Hashim *et al* showed that co-administration of metformin and CC had better results compared with NAC and CC in increasing rates of ovulation and pregnancy and even in reducing the abortion rate. As well as in patients receiving metformin and CC, estrogen and progesterone levels and also endometrial thickness were at a high level on the day of hCG administration (27).

A prospective randomized placebo-controlled pilot study on 60 Iranian women with PCOS (aged 25-35 yr) undergoing intracytoplasmic sperm injection showed that NAC (1800 mg) improves oocyte and embryo quality and could be administered as an alternative to metformin (28). Also, NAC as an adjuvant to CC for induction of ovulation improves ovulation and pregnancy rates in PCOS patients with beneficial impacts on endometrial thickness (29). A systematic review of clinical trials showed that antioxidants and vitamins have positive effects on the management of PCOS women. Although it seems more studies is necessary for this field (30).

According to the results of this study and other research, we can conclude that administration of NAC in CC- resistant PCOS patients has no effect on follicles maturation, and endometrial thickness. Some studies have suggested that reduced insulin resistance is a mechanism for NAC performance. On the other hand, since metformin mechanism is similar with NAC, it seems that different results on the combined use of NAC with these two drugs CC and letrozole are affected by other factors such as genetic factors, dosage, and duration of drug use and the sample size.

So, further studies are needed to be done on NAC therapy effects with a larger sampling in the large geographical areas and assessment of NAC therapy effects on biomedical, hormonal and metabolic profiles, symptoms of hyperandrogenism, and cardiovascular risk factors. However, our study was limited to one treatment cycle, raising the dosage of NAC will further increase the cost, which is an important consideration in choosing the appropriate therapy.

## Conclusion

NAC is not effective in inducing or augmenting ovulation in PCOs patients who were candidates for IUI and cannot be recommended as an adjuvant to CC in such patients.

## Note

***Registration ID in RCT: IRCT20160300826962N1***
